# Orthopedic Oncology – “The Challenges Ahead”

**DOI:** 10.3389/fsurg.2014.00027

**Published:** 2014-07-21

**Authors:** Ajay Puri

**Affiliations:** ^1^Department of Orthopaedic Oncology, Tata Memorial Hospital, Mumbai, India

**Keywords:** sarcoma, evidence, training, cost-effective, oncology

Orthopedic oncology or the art and science of management of musculoskeletal tumors is one of the relatively newer sub specialties in orthopedics.

The last few decades have seen rapid strides in the field of musculoskeletal oncology with amputation no longer remaining the only option to achieve local control in malignant bone tumors. Function preserving alternatives in these lesions have now become the norm without compromising on overall disease survival and have resulted in a documented improvement in overall quality of life of patients (1). As surgeons, our goal posts have now changed and the challenge has shifted. It is no longer limited to just resection of disease and restoration of function but includes being able to go about this in the most affordable manner. There is increasing pressure for medical technology assessment to include cost-effectiveness analyses to help determine difficult resource allocation decisions (2).

Additionally, there is also a pressing need to create awareness about these uncommon lesions, rapidly disseminate and propagate current information and techniques, train care givers from diverse geographical backgrounds, set up collaborative networks to gain further insight into these rare lesions and help develop evidence based protocols.

More than 80% of the population of the world is located in developing countries where there exists considerable diversity in terms of resource and expertise availability when it comes to managing musculoskeletal lesions (3). The gamut ranges from centers that offer the latest technological advances to surgeons forced to resort to amputations due to infrastructural hurdles (4). Modern orthopedics is expensive, and even personnel trained to the highest theoretical and practical level will have to continuously innovate and improvise when confronted with economic constraints (3). The absence of structured training programs and opportunities in developing countries results in a paucity of trained musculoskeletal oncologists (5). It is little wonder that a large number of patients in some of these areas are treated by inexperienced surgeons without observing oncologic principles (6).

Various exchange initiatives where experienced surgeons travel to resource challenged areas to share their knowledge and expertise serves as an excellent example of a symbiotic relationship (7). The “expert” helps the onsite medical team update their knowledge and skills while stepping outside his “comfort zone” and being forced to work with locally available resources (8). This experience often results in development of innovations, which with further refinements provide cost-effective acceptable alternative solutions globally. The various “musculoskeletal” and “bone tumor” societies and associations need to strive to promote such interactions by serving as a bridge between the “trainer” and the “trainees” (9).

Early diagnosis helps in achieving improved outcomes for patients with cancer (10). Delays in diagnosing bone and soft tissue sarcomas are frequent both because of their rarity and because the clinical features may be confused with other conditions. There is a need to educate and create awareness about these lesions. Unfortunately, even among countries with well developed health care networks there is dichotomy in data from various centers regarding the impact that awareness campaigns and education have had on early presentation and referral of bone and soft tissue sarcomas. While Denmark demonstrated that cancer patient pathways have accelerated the diagnostic process for sarcomas with a reduction in tumor size, the UK showed almost no difference in size at presentation over time (11, 12). There is considerable room for improvements in this sphere with an urgent need for new strategies.

Alvin Toffler hits the nail on the head when he says “*The illiterate of the 21st century will not be those who cannot read and write, but those who cannot learn, unlearn, and relearn*.” Over the years, many beliefs and practices become entrenched as tried and tested, and we believe they are based on scientific evidence (13). While today’s “new age medicine” expounds the necessity of tangible evidence for every clinical decision, the truth remains that observational studies dominate orthopedic surgery literature (and most medical literature) forming the basis for most therapeutic decisions including those for bone tumors (14).

While the importance of clinical expertise and experience is unquestionable, we do need to combine this with the judicious integration of best available scientific evidence to facilitate rational “informed” clinical decision making. The issue of antibiotic prophylaxis and post treatment surveillance in bone tumors highlight just two examples in orthopedic oncology where guidelines based on evidence are lacking.

In a systematic review to determine infection rates following endoprosthetic reconstruction in long bone tumors the duration of antibiotic prophylaxis ranged from “intraoperative dosing only” to “ >72 h.” Current clinical practice is highly varied with respect to antibiotic duration and there is a need to have evidence based guidelines, not only to stream line escalating healthcare costs but also prevent antibiotic misuse and overuse, which lead to antibiotic resistance, an issue of increasing clinical importance. The multicentre randomized control trial coordinated by the Center for Evidence Based Orthopaedics at McMaster University to determine the optimal dosing regimen among patients undergoing surgical excision and endoprosthetic reconstruction of bone tumors is a commendable step to try and fill this lacuna (15).

Modern multimodality therapy has improved patient survival; hence, follow-up surveillance strategies are becoming increasingly important with significant clinical and fiscal implications. Whether an increased frequency of follow-up visits and the use of various expensive imaging modalities for screening and early detection of recurrence actually results in improving overall survival of patients with sarcomas is a question that remains as yet unanswered. The financial costs incurred by surveillance are considerable and this includes both the cost to the health service and to the patient in terms of hospital visits and lost working days. Ideally, any follow-up program should be able to prove that its benefits and savings exceed its risks and costs. Although guidelines have been suggested for follow-up of patients with sarcomas, there is a paucity of data in medical literature on the effectiveness of these recommendations. There is reasonable evidence in literature from other solid tumor types, i.e., breast cancer, colorectal cancer, endometrial cancer, and melanoma that challenges the usefulness of multiple follow-up imaging tests in terms of efficacy, cost-effectiveness, and survival benefit and recent publications seem to support the same surmise for bone and soft tissue sarcomas (16, 17).

In an increasingly “cost conscious” health care scenario, allocation of limited health funding is best guided by these newer evidence based recommendations rather than empirical beliefs. We need to rapidly develop the ability to “unlearn and relearn” or else risk being condemned as “illiterate.”

It is this ability to question, reason, continually innovate, and find sustainable solutions that distinguishes us from our primate ancestors. These are qualities that we must encourage and inculcate in our colleagues and trainees. We need to stimulate them to break free from the constraints of hierarchical acceptance of practices steeped in convention without solid scientific merit. That would be one of the best legacies that we could bestow on future generations.

## Conflict of Interest Statement

The author declares that the research was conducted in the absence of any commercial or financial relationships that could be construed as a potential conflict of interest.
